# Spatial TIME landscape and its prognostic value in the lung and brain tumor: location matters

**DOI:** 10.1038/s41392-023-01473-w

**Published:** 2023-05-08

**Authors:** Yunke Wang, Anne E. Geller, Jun Yan

**Affiliations:** 1 Division of Immunotherapy, The Hiram C. Polk, Jr., MD Department of Surgery, Immuno-Oncology Program, Brown Cancer Center, University of Louisville School of Medicine, Louisville, KY USA; 2 Department of Microbiology and Immunology, University of Louisville School of Medicine, Louisville, KY USA

**Keywords:** Cancer microenvironment, Tumour immunology

**Fig. 1:**
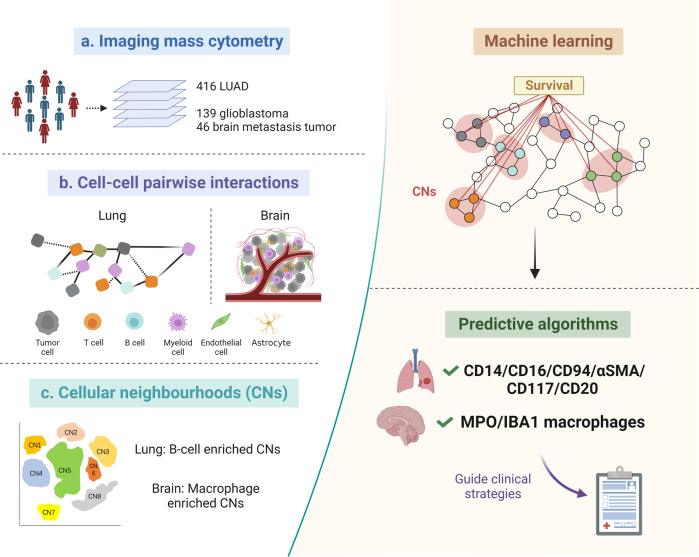
The spatial architecture of the TIME predicts clinical outcomes. **a** Researchers applied imaging mass cytometry (IMC) to generate multiplexed images of lung tumors from 416 lung adenocarcinoma (LUAD) patients, as well as brain tumors from 139 high-grade glioma and 46 brain metastasis patients. **b** Cellular interaction analysis revealed spatial interactions among tumor cells, T cells, B cells, myeloid cells, endothelial cells, and astrocytes in lung and brain tumors. **c** Correlating cellular neighbourhood (CN) patterns with overall survival (OS). B cell-enriched CNs were associated with increased OS in LUAD. Long-term survival (LTS) patients with glioblastoma showed high macrophage-enriched CNs. Machine learning of spatial characteristics from lung and brain tumors established two sets of clinically relevant predictive immune markers: CD14, CD16, CD94, αSMA, CD117 and CD20 for LUAD; MPO and IBA1 for brain tumors. Schematics were created using BioRender

In two recently published papers in *Nature*,^1,2^ Logan Walsh and colleagues utilized imaging mass cytometry (IMC) to create comprehensive single-cell spatial landscapes of the tumor immune microenvironment (TIME) in lung tumors from 416 lung adenocarcinoma (LUAD) patients, as well as brain tumors from 139 high-grade glioma and 46 brain metastasis (BrM) patients. By conducting a thorough analysis to characterize cellular spatial interactions and neighborhood patterns, and correlating these spatial features with clinical outcomes (Fig. 1), the researchers gained novel insights into the complexities of tumoral architecture. Ultimately, they identified two sets of clinically relevant predictive immune markers that could be used to assist in guiding clinical decisions.

In lung cancer samples, correlation analysis between immune cell frequencies and clinical variables revealed that B cells were an independent predictor of improved survival. To gain broader spatial insight into the TIME of LUAD, the likelihood of cellular interaction and avoidance behaviors were quantified via cell-cell pairwise interaction analyses. High-grade tumors exhibited increased neutrophil and endothelial cell interactions with cancer cells, increased CD8^+^ T cell and B cell interactions with CD163^+^ macrophages, and weaker CD8^+^ and CD4^+^ T cell interactions with cancer cells, characterizing a higher tendency for metastasis and an immunosuppressive TIME formation. They next correlated cellular phenotype with survival. Ki-67^+^ endothelial cells and HIF-1α^+^ neutrophils were related to worse overall survival (OS), whereas CD4^+^ T cells with activated ERK signaling, which inhibited regulatory T cell (Treg) differentiation, were related to increased OS, suggestive of a spatial association between aggressive tumors and hypoxia and immunosuppression.

As anticipated, the brain TIME was dominated by astrocytes, macrophages, and infrequent lymphocytes. Compared to glioblastoma, BrM possessed higher immune infiltration, particularly melanoma BrM, explaining the potential responsiveness to immune checkpoint blockade therapy. BrMs also exhibited distinct vasculature distribution preferences. Endothelial cells showed strong interactions with cancer cells in both settings, which was essential for metastatic colonization and invasion of the Blood Brain Barrier (BBB). Perivascular cancer cells and monocyte-derived macrophages (MDMs) in glioblastomas exhibited lower proliferation. The perivascular enriched CD40^+^ M1-like and OX40L^+^ M2-like MDMs recruited more helper T (T_h_) cells, establishing a vascular micro-niche that modified the brain TIME to restrain cancer cell expansion. In contrast, endothelial cells that interacted with cancer cells in BrM showed higher proliferation but were suppressed by CD8^+^ T cell-interactions, specifically in BrM cores. Cancer-adjacent endothelial cells in BrM cores showed lower expression of tight junction protein claudin-5, resulting in weaker endothelial junctions and peritumoral oedema. These studies on interacting cellular programs reveal underlying spatially organized immune-cancer cell networks that illuminate the basic biology of tumor progression and metastasis.

To explore in-depth analysis of the spatial architecture of the TIME, researchers next identified cellular neighborhoods (CNs) by defining two variables: the interacting cell numbers within a CN and the total number of CNs, and correlated CN frequencies with clinical survival by Kaplan-Meier analysis. In LUAD, B cell-enriched CNs were associated with increased OS after exclusion of B/T cell interaction bias. The association could be negated by Treg cell enrichment, but further improved through the interaction of B cells and CD4 helper T cells, illustrating the prognostic value of B cell spatial communities in lung cancer. In brain tumors, M1-like MDM enriched CNs were associated with increased survival, likely due to interactions with neutrophils and M1-like microglia. However, no such correlation existed between the frequency of these cells and survival. Compared to short-term survival (STS), long-term survival (LTS) patients with glioblastoma showed enrichment of a CD163^-^P2Y12^-^ M1-like MDM population. Together, these studies show that multicellular spatial neighborhood patterns, not abundance alone, provide critical prognostic information that can help establish predictive algorithms for clinical strategies.

Stage I LUAD patients do not receive adjuvant chemotherapy according to best-practice clinical guidelines; however, they often relapse after surgical resection. To predict progression in early-stage LUAD patients, researchers established a deep-learning approach by inputting raw LUAD IMC images into the Resnet50 neural network model for feature extraction. Machine learning of spatial IMC data from specific lineage markers enhanced the prediction accuracy of progression in a cohort of 286 stage I NSCLC patients far beyond correlations using clinical variables and cell frequencies. Using CNs, a panel of five markers including CD14, CD16, CD94, αSMA, and CD117 was identified and validated in a cohort of 60 LUAD patients with 90.8% prediction accuracy. Adding CD20 further increased prediction accuracy to 93.3% with 95.6% precision and recall.

In glioblastoma, macrophage clusters enriched in LTS highly expressed MPO. Compared to MPO^-^ macrophages, neutrophil-like MPO^+^ macrophages were characterized by high expression of GMP-derived lineage-related signatures *S100A8* and *S100A9*, cytotoxicity-related reactive oxygen species biosynthesis, phagosome formation, and signatures of HIF-1α signaling, indicting an enhanced anti-tumor phenotype. High densities of MPO^+^ macrophages increased the frequency of innate effectors, enhanced spatial interactions between endothelial cells and M1-like MDMs with T cells, and reduced interactions between M2-like MDMs and CD8^+^ T cells, ultimately polarizing the regional TIME towards anti-tumor functionality. As the majority of MPO^+^ macrophages were CD163^-^P2Y12^-^ and were associated with better survival, they chose the combination of MPO and IBA1 as the predictive algorithm for clinical outcomes in glioblastoma.

These two remarkable works represent a groundbreaking paradigm for utilizing single-cell spatial features encoded in multiplex IMC images to classify clinical outcomes. Recently, several studies have addressed the significance of deciphering solid tumor landscapes at a single-cell spatial resolution. Spatial profiling, for instance, aids in establishing a spatial single-cell pathology to better differentiate patients with distinct clinical outcomes in breast cancer,^3^ and can be utilized to predict response to immunotherapy in melanoma.^4^ The 1-mm^2^ sample size of IMC also reduces biopsy-related trauma while maintaining prediction accuracy. The minimal tissue requirement and clinically relevant markers make it possible to translate spatial TIME information into predicting prognoses and response to treatments, thus paving the way for precision medicine. However, the highly heterogeneous TIME may present a challenge in interpreting these analyses for clinical decision-making, and future studies are warranted.
